# Inspiratory muscle training after atrial fibrillation catheter ablation: A pilot study

**DOI:** 10.1097/MD.0000000000043181

**Published:** 2025-07-04

**Authors:** Gaku Oguri, Eisuke Amiya, Katsuhito Fujiu, Masanobu Taya, Tsukasa Oshima, Yu Shimizu, Kenichiro Yamagata, Eriko Hasumi, Toshiya Kojima, Norihiko Takeda

**Affiliations:** a Department of Cardiovascular Medicine, Graduate School of Medicine, The University of Tokyo, Tokyo, Japan; b Department of Advanced Cardiology, Graduate School of Medicine, The University of Tokyo, Tokyo, Japan.

**Keywords:** atrial fibrillation, cardiac rehabilitation, catheter ablation, inspiratory muscle training

## Abstract

Atrial fibrillation management is challenging, and traditional cardiac rehabilitation often overlooks respiratory issues. We aimed to evaluate the safety and efficacy of cardiac rehabilitation with inspiratory muscle training to enhance respiratory and cardiac functions after atrial fibrillation catheter ablation. This prospective, single-center observational study, conducted at the University of Tokyo Hospital between February 2019 and January 2020, included 5 men (average age 68.4 ± 2.58 years) who underwent initial ablation for symptomatic paroxysmal (n = 1) or non-paroxysmal (n = 4) atrial fibrillation. The participants underwent inspiratory muscle training alongside standard cardiac rehabilitation (intensity: 20% of the maximal inspiratory pressure, adjusted weekly). The pre- and post-intervention ejection fraction, left atrial volume index, and brain natriuretic peptide levels were evaluated. Inspiratory muscle training integration was associated with improvements in respiratory muscle strength and pulmonary function. The average ejection fraction improved from 62.0% to 64.4%, the left atrial volume index decreased from 39.4 mL/m^2^ to 27.0 mL/m^2^, and brain natriuretic peptide levels reduced from 112.28 pg/mL to 20.98 pg/mL. The anaerobic threshold increased from a mean of 12.3 to 14.2, and the mean peak oxygen uptake increased from 16.72 mL/kg/min to 18.12 mL/kg/min. Over a 4-year follow-up, atrial fibrillation recurrence was observed in only 1 of the 5 patients. Inspiratory muscle training, when integrated with cardiac rehabilitation, could potentially improve respiratory and cardiac function in patients with post-atrial fibrillation ablation and may help reduce the likelihood of atrial fibrillation recurrence. This pilot study supports the potential of inspiratory muscle training in enhancing standard rehabilitation protocols, warranting further investigation in larger randomized trials to substantiate these findings and explore long-term benefits.

## 1. Introduction

Atrial fibrillation (AF) is the most common cardiac arrhythmia, affecting 1% to 2% of the global population and posing substantial challenges in cardiovascular medicine.^[1]^ AF pathophysiology involves complex interactions between electrical, structural, and autonomic factors in the atria, influenced by aging, hypertension, obesity, and genetic predisposition.^[2]^ Catheter ablation, particularly pulmonary vein isolation, has become a cornerstone of AF management.^[3]^ However, the long-term success of ablation, especially in cases of persistent AF, is limited by arrhythmia recurrence.^[4]^ Traditional cardiac rehabilitation (CR) programs focus on restoring cardiovascular function through aerobic exercise, lifestyle education, and stress management. However, these programs often overlook specific respiratory challenges faced by patients with AF. Improved pulmonary function can significantly influence cardiac health, particularly in AF management, where efficient breathing mechanics can stabilize heart rhythms and enhance the overall cardiac output. However, current rehabilitation protocols often lack targeted interventions to strengthen respiratory muscles, which is critical for patients recovering from procedures such as catheter ablation. This gap highlights the need for targeted respiratory interventions to complement the cardiovascular focus of traditional rehabilitation. Recent advancements in ablation technology, including cryoballoon and pulsed-field ablation, have improved procedural safety and efficacy; however, recurrence rates post-ablation emphasize the need for comprehensive post-procedural care.^[5–7]^ Research on AF ablation and respiratory function has reported that cryoballoon ablation for paroxysmal AF can mildly improve lung function.^[8]^

Furthermore, studies have highlighted the critical role of respiratory function in the quality of life and prognosis of patients with AF, showing that respiratory muscle strength, pulmonary function, and functional capacity directly impact AF outcomes.^[9]^ These considerations are even more relevant for patients with conditions like chronic obstructive pulmonary disease (COPD), where diminished respiratory function is independently associated with worse AF treatment outcomes.^[10]^ Inspiratory muscle training (IMT) improves respiratory function in patients with respiratory diseases such as COPD. IMT has also shown promising effects in patients with cardiovascular conditions, including AF and heart failure. IMT can improve pulmonary function, respiratory muscle strength, and functional capacity in patients with AF.^[11]^ In heart failure, IMT has been associated with improved exercise capacity, quality of life, and cardiorespiratory responses.^[12]^

Additionally, IMT reduces blood pressure and sympathetic activity in patients with hypertension. The benefits of IMT extend to individuals with reduced and preserved ejection fraction (EF).^[13]^ The benefits of IMT, including enhanced respiratory muscle performance and autonomic modulation, are crucial for patients with post-AF ablation, where respiratory efficiency can influence cardiac stability and the likelihood of arrhythmia recurrence.

By highlighting the benefits of respiratory muscle training, this study could set a new standard for post-ablation care, contributing to more holistic clinical practices for patients with AF. To our knowledge, no previous studies have investigated the effects of adding IMT to CR specifically in a post-AF ablation population, making this study novel and necessary. Furthermore, our findings underscore the potential for IMT to be implemented as a routine component of post-ablation care, potentially driving a paradigm shift in the management of AF patients. This pilot study aimed to integrate IMT with standard CR protocols to evaluate the safety and efficacy of this combination in enhancing respiratory and cardiac functions in patients with post-AF catheter ablation. The objective of this study was to assess the effects of adding IMT to CR on respiratory and cardiac recovery post-ablation. By integrating IMT, we aim to improve immediate post-procedure outcomes and establish a foundation for sustained improvements in patients’ cardiovascular health.

## 2. Materials and methods

### 2.1. Statement of human rights

The study was approved by the University of Tokyo Ethics Review Board (approval number: 10888) and was performed in accordance with the principles of the Declaration of Helsinki [name of the Ethics Committee: Tokyo University Graduate School of Medicine and Faculty of Medicine Ethics Committee A; the Chairperson of the Ethics Committee: Akabayashi Akira; protocol number: 10888-(1); date of approval: August 1, 2016]. This manuscript complies with the STROBE guidelines for observational studies. We have included a completed STROBE checklist in the Supplementary Materials, Supplemental Digital Content, https://links.lww.com/MD/P323, to confirm our adherence to these reporting standards.

### 2.2. Patient consent

Informed consent was obtained from all individual participants included in the study.

### 2.3. Study population

This prospective, single-center observational study included 5 consecutive patients who underwent initial ablation for symptomatic paroxysmal AF (PAF; n = 1) or non-PAF (n = 4) at the University of Tokyo Hospital between February 2019 and January 2020. Patients aged ≥ 20 years who had only undergone initial AF treatment, provided informed consent, and could undergo respiratory function tests were included. Patients on dialysis, those who had undergone lung surgery or treatment for lung cancer, or those with active pneumonia were excluded. All methods were performed in accordance with relevant guidelines and regulations, including the 2017 HRS/EHRA/ECAS Expert Consensus Statement on Catheter and Surgical Ablation of Atrial Fibrillation: recommendations for patient selection, procedural techniques, patient management and follow-up, definitions, endpoints, and research trial design. The non-PAF group included patients with persistent AF (defined as episodes persisting for > 7 days and not self-terminating) and those with long-standing persistent AF (continuous AF events extending for > 12 months).

Catheter ablation was prescribed to patients with symptomatic PAF and non-PAF at a physician’s discretion. The procedures were performed under general anesthesia after obtaining written informed consent. All patients consented to participate in CR. The University of Tokyo Ethics Review Board reviewed and approved this study (approval number: 10888). The study protocol adhered to the Declaration of Helsinki.

### 2.4. Ablation protocols

Ablation methods for the treatment of patients with PAF were chosen based on the 2017 HRS/EHRA/ECAS/APHRS/SOLAECE Expert Consensus Statement on Catheter and Surgical Ablation of Atrial Fibrillation^[14]^ and 2012 HRS/EHRA/ECAS Expert Consensus Statement on Catheter and Surgical Ablation of Atrial Fibrillation: Recommendations for patient selection, procedural techniques, patient management and follow-up, definitions, endpoints, and research trial design. Only the radiofrequency catheter ablation (RFCA) method was used to treat patients without PAF. A single trans-septal puncture was performed after accessing the arteries and veins. Pulmonary vasculature (PV) angiography potential measurements were performed before and after PV isolation using circular mapping catheters. A 3-dimensional navigation system (Carto-3; Biosense Webster, Inc., Irvine) was used to perform RFCA, with a Thermocool ablation catheter (Biosense Webster, Inc.) delivering point-by-point radiofrequency pulses at 35 to 50 W. In cryoballoon ablation, a 28-mm cryoballoon (ARC-Adv-CB, Arctic Front Advance; Medtronic, Inc., Minneapolis) with an inner lumen mapping catheter (Achieve, Medtronic) was inflated and introduced into each PV orifice through a steerable 15F sheath (FlexCath Advance; Medtronic). Optimal PV occlusion was achieved using contrast injection before applying cryothermal energy for 180 seconds, followed by 120 seconds. A decapolar catheter was introduced into the superior vena cava, and the phrenic nerve was paced continuously during cryothermal energy application to the right superior and inferior PVs at a cycle length of 1000 ms, current of 25 mA, and pulse width of 2 ms. The diaphragmatic excursion was assessed through palpation, and diaphragmatic compound motor action potentials were monitored to prevent phrenic nerve injury. Compound motor action potentials were recorded, as previously reported. Cryothermal energy application was discontinued when diaphragmatic excursions decreased on palpation or when the compound motor action potential amplitude was reduced by >30%. All patients received anticoagulant therapy for 4 weeks before ablation and for at least 6 months after ablation. Antiarrhythmic drugs were suspended for 1 week before ablation. The anticoagulant therapy was not interrupted perioperatively. Heparin was administered intraoperatively to maintain an activated clotting time of 300 to 350 seconds.

### 2.5. CR

The CR program included anaerobic ergometer exercises and low-intensity limb resistance training. The exercise intensity was individualized based on the anaerobic threshold (AT) identified via cardiopulmonary function testing. Generally, the exercise sessions began at 10 minutes and gradually increased to 30 minutes, with vital signs monitored every 10 minutes by medical staff. The recommended frequency of these sessions was at least 3 times per week. The CR program was conducted following the JCS/JACR 2012 Guidelines on Rehabilitation in Patients with Cardiovascular Disease. CR commenced 1 month after ablation.

### 2.6. IMT

IMT was initiated concurrently with aerobic training using a variable flow-resistive loading device (POWERbreatheKH1; HaB Direct, Southam, UK). The initial IMT intensity was set at 20% of the maximal inspiratory pressure and included 2 sets of 30 repetitions daily. The training frequency was maintained at 7 days per week, with a gradual increase in intensity. Follow-up measurements of maximal inspiratory pressure were performed weekly. These measurements were used to adjust the training load in the subsequent weeks. Additionally, the Borg scale (ratings of perceived inspiratory effort ranging from 4 to 6 out of 10) was used to guide decisions regarding increments in the training load. IMT was initiated simultaneously with the CR program.

### 2.7. Clinical assessments

Brain natriuretic peptide (BNP) levels were assessed before and 6 months after ablation. The rationale for this timing was based on the structure of the outpatient CR program, which concluded after 5 months, marking the 6-month point as a period of stabilized rehabilitation. Additionally, this study integrated ablation with CR and IMT as a unified approach, warranting assessment at 2 key time points: pre-ablation and 6 months post-ablation. BNP was chosen because it is a widely used biomarker for the clinical evaluation of cardiac function.

### 2.8. Respiratory function tests

Respiratory function tests were performed before and 6 months after ablation using the FUDAC-7C (FUKUDA DENSHI Co., Ltd., Tokyo, Japan). All spirometric reference values were based on the Japanese Respiratory Society Pulmonary Physiology Committee (JRS 2001).^[15]^ The spirometric parameters included vital capacity (VC; the volume of air breathed out after the deepest inhalation), forced vital capacity (FVC), and forced expiratory volume in 1 seconds (FEV_1_).

### 2.9. Data analysis

Variables were expressed as means ± standard deviation or numbers and percentages, as appropriate. The associations between lung function variables and demographic and clinical parameters were analyzed using paired-sample *t*-tests. Statistical significance was set at *P* < .05. All analyses were performed using SPSS statistical software version 26 (IBM Corp., Armonk).

## 3. Results

### 3.1. Patient characteristics

Our study included 5 patients who underwent initial ablation for symptomatic PAF or non-PAF at the University of Tokyo Hospital between February 2019 and January 2020. The baseline characteristics of the patient cohort are summarized in Table 1. The average age of participants was 68.4 years. All participants were diagnosed with different forms of AF: 1 with PAF and the other with non-paroxysmal forms, including long-standing AF and persistent AF.

**Table 1:** Patient characteristics.

Age (yr)	68.4 ± 2.58
Sex	Men (5)
Procedure	RF (4) CBA (1)
AF	PAF (1) PeAF (2) LSAF (2)
BMI (kg/m^2^)	23.22 ± 1.86
EF (%)	62 ± 8.66
LAVi	39.4 ± 10.81
CHA2DS2-VASc	2 ± 0.894
Smoker	(0/5)
Cr (mg/dL)	0.762 ± 0.040
DM	(3/5)
BA, COPD	(0/5)
HTN	(2/5)
BNP (pg/dL)	35.72 ± 28.0

AF = atrial fibrillation, BA = bronchial asthma, BMI = body mass index, CBA = cryoballoon ablation, CHA2DS2-VASc = congestive heart failure, hypertension, age, diabetes, previous stroke/transient ischemic attack, sex category-vascular disease, COPD = chronic obstructive pulmonary disease, Cr = creatinine, DM = diabetes mellitus, EF = ejection fraction, HTN = hypertension, LAVi = left atrial volume index, LSAF = long-standing AF, PAF = paroxysmal atrial fibrillation, PeAF = persistent AF, RFCA = radiofrequency catheter ablation.

The generalized characteristics of the patient cohort included an average body mass index of 23.22 ± 1.86 kg/m^2^, indicating a normal weight range for all participants. Cardiac function, as measured by EF, was maintained within a relatively normal range across the cohort, averaging 62 ± 8.66%. The left atrial volume index (LAVi), an indicator of left atrial size, was averaged at 39.4 ± 10.81 mL/m^2^. The mean CHA2DS2VASc score, a clinical prediction tool for estimating the risk of stroke in patients with AF, was 2 ± 0.894. Additionally, the patients were not current smokers, indicating no immediate tobacco-related risks.

The average creatinine level was 0.762 ± 0.040 mg/dL, which falls within the normal range. Comorbid conditions such as diabetes mellitus and hypertension were present in 3 and 2 of the 5 patients, respectively, highlighting the typical comorbidity profile seen in AF populations. Baseline BNP levels, a marker for cardiac dysfunction, averaged 112.28 pg/mL, suggesting variance in cardiac stress among the patients.

### 3.2. Adverse events during exercise

Every exercise was performed using electrocardiogram monitoring, and the patients’ rhythms were checked during the training. No significant adverse events were recorded, confirming the safety of the combined CR with IMT in patients undergoing post-AF ablation. No cases of new-onset arrhythmias (such as AF or ventricular tachycardia), exacerbations of heart failure, or side effects were identified during exercise training.

### 3.3. Clinical and functional outcomes

Clinical assessments were conducted to evaluate the changes in respiratory and cardiac parameters before and after the intervention. The following outcomes highlight the overall changes observed:

EF: Mean EF improved from 62.0% pre-intervention to 64.4% post-intervention.
LAVi: Mean LAVi reduced from 39.4 to 27.0, indicating improved atrial compliance.
BNP: BNP levels significantly decreased, from an average of 149.2 pg/mL pre-treatment to 20.98 pg/mL post-treatment, indicating a reliable reduction in cardiac stress. These changes are presented in Table 2.

**Table 2:** Summary of clinical and functional outcomes.

Parameter	Pre-intervention mean (SD)	Post-intervention mean (SD)	*P*-value
Ejection fraction (%)	62.0 (5.77)	64.4 (5.50)	.329
Left atrial volume index (mL/m^2^)	39.4 (11.62)	27.0 (7.05)	.155
Brain natriuretic peptide (pg/mL)	149.2 (69.5)	21.00 (18.87)	.011

### 3.4. Pulmonary function and respiratory muscle strength

VC increased from an average of 96.9% to 101.9% of the predicted values.

FVC showed a slight improvement from 97.5% to 101.8% of the predicted values. FEV_1_ remained stable, with only a minimal change from 73.8% to 73.9%. These outcomes are summarized in Table 3.

**Table 3:** Pulmonary function, respiratory muscle strength, and cardiopulmonary exercise testing

Parameter	Pre-intervention mean (SD)	Post-intervention mean (SD)	*P*-value
Vital capacity (VC)	96.9 (8.36)	101.9 (10.12)	.139
Forced vital capacity (FVC)	97.5 (9.21)	101.8 (9.76)	.208
Forced expiratory volume in 1 second (FEV_1_)	73.8 (6.79)	73.9 (6.22)	.98
AT (mL/kg/min)	12.3 (1.92)	14.28 (2.13)	.039
Peak VO_2_ (mL/kg/min)	97.5 (9.21)	101.8 (9.76)	.208

AT = anaerobic threshold, FEV_1_ = forced expiratory volume in 1 second, FVC = forced vital capacity, VC = vital capacity, Peak VO_2_ = peak oxygen uptake.

### 3.5. Cardiopulmonary exercise testing

**Figure 1.**
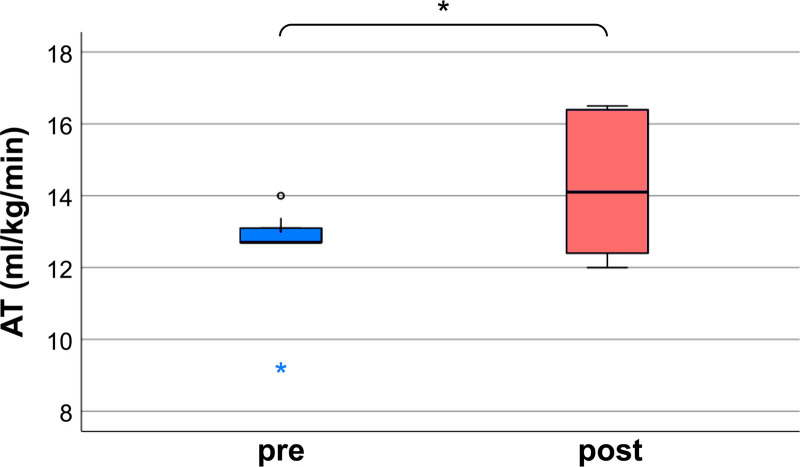
AT changes. The boxplot shows the distribution of AT values before and after the intervention. *The paired *t*-test resulted in a *P*-value of .039, indicating a significant improvement in AT after the rehabilitation program. AT = anaerobic threshold.

**Figure 2.**
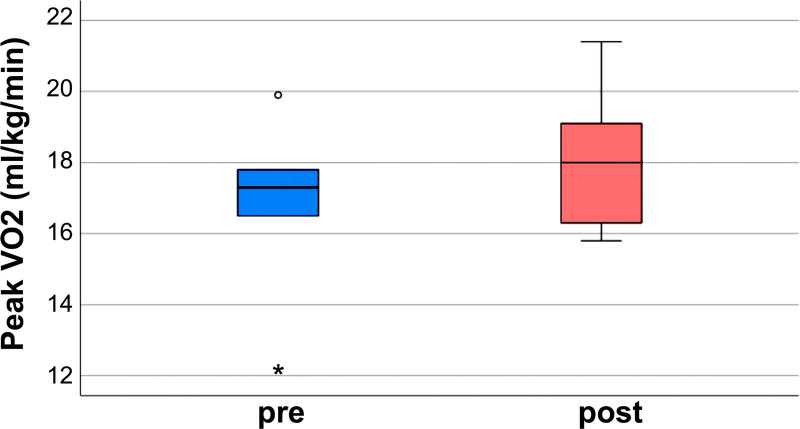
VO_2_ changes. The boxplot illustrates the changes in peak VO_2_ values. The paired *t*-test yielded a *P*-value of .111, indicating no post-intervention improvement in aerobic capacity. VO_2_ = peak oxygen uptake.

AT increased from a mean of 12.3 to 14.2, reflecting enhanced exercise tolerance. This improvement is illustrated in Figure 1 and Table 3. Peak oxygen uptake (peak VO_2_): The mean peak VO_2_ increased from 16.72 mL/kg/min to 18.12 mL/kg/min, demonstrating improved aerobic capacity. This observation is also detailed in Figure 2 and Table 3.

## 4. Discussion

This study substantiated the established link between respiratory dysfunction, such as COPD, and the elevated risk of developing AF. Our findings indicate that IMT might offer potential benefits beyond traditional respiratory applications, possibly contributing to improvements in cardiac and respiratory functions, even in patients without COPD. The observed stability in FEV_1_, despite improvements in VC and FVC, highlights the selective enhancement of inspiratory muscle function rather than expiratory capabilities. This selective improvement, which is often overlooked in traditional CR programs, may specifically reduce respiratory workload and enhance oxygen intake efficiency. Such improvements could be particularly beneficial for patients with compromised cardiovascular health. Furthermore, the increase in AT from 12.3 to 14.2 indicates enhanced exercise tolerance, suggesting patients may sustain higher physical activity levels without experiencing anaerobic metabolism. This could potentially reduce exercise-induced arrhythmias in AF patients. Although the increase in peak VO2 was not statistically significant, the trend toward improved aerobic efficiency supports the hypothesis that strengthening respiratory muscles can positively influence overall cardiac function. This broad applicability suggests that IMT could be a critical component in managing diverse populations with varying cardiovascular risks. Patients with COPD are at a 28% to 74% higher risk of developing AF, escalated by factors such as frequent exacerbations, enlarged left atrium, or elevated systemic inflammatory levels.^[16]^ These factors contribute to chronic hypoxia, systemic inflammation, and altered thoracic pressures that promote atrial remodeling – the primary pathological process leading to AF.^[17]^ These insights emphasize the critical need for the proactive management of respiratory dysfunction to improve outcomes in patients with AF, suggesting that interventions focusing on respiratory health could play a pivotal role in AF management. Expanding the use of IMT in routine clinical practice could address the immediate needs of AF patients and contribute to the broader strategy of heart disease prevention and management. IMT is increasingly recognized for its broad applicability and benefits, which extend beyond patients with existing chronic respiratory conditions. IMT enhances inspiratory muscle strength and endurance, thereby improving pulmonary ventilation and increasing oxygen saturation. This improvement is crucial, as it directly reduces the cardiac workload, particularly during physical exertion, and indirectly mitigates the burden of AF by stabilizing the autonomic nervous system. The reduction in sympathetic overdrive, a common trigger for AF, further illustrates how enhanced respiratory function can contribute to cardiac stability.^[10,11,18]^ Implementing IMT across a wide range of clinical scenarios could redefine standard care protocols, promoting a more integrated approach to treating AF and other related cardiovascular disorders. In recent reports, IMT has delivered benefits for patients undergoing cardiac operations and those with ischemic heart disease, especially by enhancing respiratory muscle strength and reducing hospital stays and quality of life.^[19–21]^

The implementation of IMT in CR programs may have potential in enhancing pulmonary function and could potentially improve overall cardiac function. Our study observed notable improvements in key cardiac parameters, such as EF and LAVi, in participants undergoing IMT.

These findings suggest that IMT might have the potential to help stabilize cardiac rhythm and possibly reduce AF recurrence. The broader implications of these benefits are supported by additional research demonstrating that IMT can improve cardiorespiratory responses and increase exercise capacity in cardiac conditions, including heart failure and post-cardiac surgery.^[12,22]^ Furthermore, increased cardiorespiratory fitness is associated with reduced arrhythmia recurrence in obese individuals with AF.^[23]^ During a 4-year follow-up, only 1 of 5 patients experienced AF recurrence, highlighting the potential of IMT to improve the long-term prognosis of patients post-ablation. The presence or absence of AF recurrence was evaluated using 24-h Holter monitor electrocardiograms. This low recurrence rate supports IMT as a standard component in CR programs, emphasizing its role in enhancing respiratory and cardiac health. Furthermore, our study confirmed the safety of incorporating IMT into the rehabilitation regimen with no significant adverse events, underscoring its feasibility for widespread clinical implementation.

Despite these promising findings, our study had limitations owing to the small sample size, which restricts the generalizability of our results. Moreover, the homogeneity of the cohort, comprising solely men with varying AF types and ablation techniques, could have influenced the individual responses to the intervention. These variations highlight the need for larger and more diverse studies to validate the effectiveness of IMT in different populations and clinical settings. The promising outcomes of this pilot study advocate further research on the long-term benefits of IMT, suggesting that it may play a crucial role in preventing AF recurrence on a broader scale.

## 5. Conclusions

Given the interconnected nature of respiratory and cardiac health, integrating IMT into CR for patients with AF is a logical and potentially impactful approach. Our pilot study supports this integration, showing promising results for AF recurrence prevention through comprehensive cardiopulmonary rehabilitation. Further research in larger randomized trials is necessary to confirm these findings and refine intervention strategies, potentially setting a new standard for post-ablation care. Furthermore, identifying specific characteristics or subgroups of patients with AF for whom this program is most beneficial could enhance the overall value and applicability of the program.

## Acknowledgments

We would like to thank Editage (www.editage.jp) for English language editing.

## Author contributions

**Conceptualization:** Gaku Oguri, Eisuke Amiya, Katsuhito Fujiu.

**Formal analysis:** Gaku Oguri.

**Investigation:** Masanobu Taya.

**Project administration:** Gaku Oguri.

**Supervision:** Norihiko Takeda.

**Writing – original draft:** Gaku Oguri.

**Writing – review & editing:** Gaku Oguri, Eisuke Amiya, Katsuhito Fujiu, Tsukasa Oshima, Yu Shimizu, Kenichiro Yamagata, Eriko Hasumi, Toshiya Kojima.

## Supplementary Material


